# A phase II study of the bispecific antibody MDX-H210 (anti-HER2 × CD64) with GM-CSF in HER2+ advanced prostate cancer

**DOI:** 10.1054/bjoc.2001.1878

**Published:** 2001-07

**Authors:** N D James, P J Atherton, J Jones, A J Howie, S Tchekmedyian, R T Curnow

**Affiliations:** CRC Institute for Cancer Studies, University of Birmingham, Birmingham, B15 2TA, UK; Department of Pathology, University of Birmingham, Birmingham, B15 2TT, UK; Pacific Shores Medical Group, Long Beach, CA, 90813, USA; Medarex Inc, New Jersey, USA

**Keywords:** prostate cancer, bispecific antibody, GM-CSF, HER2, immunotherapy

## Abstract

The proto-oncogene HER2 presents a novel therapeutic target. We report results in 25 patients with HER2+ advanced prostate cancer treated with the bispecific antibody MDX-H210 15 μg m^−2^by intravenous infusion plus GM-CSF 5 μg kg^−1^day^−1^by subcutaneous injection for 4 days repeated weekly for 6 weeks. Patients with stable disease or better received further cycles of treatment until disease progression or study withdrawal. 1 patient received no treatment and 4 received less than 1 cycle and are included in the toxicity analysis only. Median duration of follow up was 105+ (range 21–188) days. Toxicity was generally NCI-CTG 0–2. There were 2 grade 4 adverse events (heart failure and dyspnoea) and 1 grade 3 event (allergic reaction) resulting in discontinuation of the study medication. There were 9 further grade 3 events not resulting in trial withdrawal. There were no treatment-related deaths. 7/20 (35%) evaluable patients had a >50% PSA response of median duration 128 (range 71–184+) days. 7/12 (58%) patients with evaluable pain had improvements in pain scores. The PSA relative velocity on therapy decreased in 15/18 (83%) assessable patients compared to pre-study. GM-CSF and MDX-H210 is active in hormone refractory prostate carcinoma with acceptable toxicity; further studies are warranted. © 2001 Cancer Research Campaign http://www.bjcancer.com


*British Journal of Cancer* (2001) **85**(2), 152–156
© 2001 Cancer Research Campaign
doi: 10.1054/ bjoc.2001.1878, available online at http://www.idealibrary.com on IDEAL®

http://www.bjcancer.com

### A phase II study of the bispecific antibody MDX-H210 (anti-HER2 × CD64) with GM-CSF in HER2+ advanced prostate cancer

**ND James[1], PJ Atherton[1], J Jones[1], AJ Howie[2], S Tchekmedyian[3] and RT Curnow[4]**

[1]CRC Institute for Cancer Studies, University of Birmingham, Birmingham B15 2TA, UK; [2]Department of Pathology, University of Birmingham, Birmingham B15 2TT, UK; [3]Pacific Shores Medical Group, Long Beach, CA 90813, USA; [4]Medarex Inc, New Jersey, USA

**Summary** The proto-oncogene HER2 presents a novel therapeutic target. We report results in 25 patients with HER2+ advanced prostate cancer treated with the bispecific antibody MDX-H210 15 $\mu$g $m^{-2}$ by intravenous infusion plus GM-CSF 5 $\mu$g $kg^{-1}$ $day^{-1}$ by subcutaneous injection for 4 days repeated weekly for 6 weeks. Patients with stable disease or better received further cycles of treatment until disease progression or study withdrawal. 1 patient received no treatment and 4 received less than 1 cycle and are included in the toxicity analysis only. Median duration of follow up was 105+ (range 21–188) days. Toxicity was generally NCI-CTG 0–2. There were 2 grade 4 adverse events (heart failure and dyspnoea) and 1 grade 3 event (allergic reaction) resulting in discontinuation of the study medication. There were 9 further grade 3 events not resulting in trial withdrawal. There were no treatment-related deaths. 7/20 (35%) evaluable patients had a >50% PSA response of median duration 128 (range 71–184+) days. 7/12 (58%) patients with evaluable pain had improvements in pain scores. The PSA relative velocity on therapy decreased in 15/18 (83%) assessable patients compared to pre-study. GM-CSF and MDX-H210 is active in hormone refractory prostate carcinoma with acceptable toxicity; further studies are warranted. © 2001 Cancer Research Campaign http://www.bjcancer.com

**Keywords**: prostate cancer; bispecific antibody; GM-CSF; HER2; immunotherapy

Prostatic cancer is the second commonest malignancy in men in developed countries and the second highest cause of cancer death. Incidence is increasing, partly due to increased rates of diagnosis due to the use of serum prostate-specific antigen (PSA) testing and partly due to the increasing age profile in the population. Tumours are initially sensitive to androgen deprivation, which can provide effective palliation of symptoms in many cases with locally advanced or metastatic disease. However, most tumours eventually become hormone resistant and further systemic therapy is usually ineffective. There is thus a pressing need for more effective therapies for this disease.

The proto-oncogene c-erbB-2/NEU/HER2, is a 185 000 MW transmembrane protein of the erbB/epidermal growth factor receptor family with intracellular tyrosine kinase activity. Inappropriate expression or expression of mutated HER2 protein results in transformation in mouse models and correlates with poor prognosis in a variety of human tumours. Overexpression of HER2 is found in 16–83% of cases of prostate cancer assessed by immunohistochemistry (Ross et al, 1993; Fox et al, 1994; Lyne et al, 1997; Morote et al, 1999) and 42–44% assessed by fluorescent in situ hybridization (FISH) (Ross et al, 1993, 1997; Kallakury et al, 1998). In addition, HER2 overexpression correlates with markers of poor prognosis in prostate cancer (Sadasivan et al, 1993; Fox et al, 1994; Ross et al, 1997; Kallakury et al, 1998; Morote et al, 1999). Recent data suggests that HER2 overexpression may functionally contribute to the development of androgen independence (Craft et al, 1999; Yeh et al, 1999).

*Received 12 April 2000*
*Revised 8 January 2001*
*Accepted 19 January 2001*

*Correspondence to:* ND James

There is increasing interest in therapeutic approaches based on HER2 following the results obtained in breast cancer therapy with Herceptin (Cobleigh et al, 1998; Slamon et al, 1998). We report preliminary results with an anti-HER2 × anti-CD64 bispecific antibody in HER2-positive hormone refractory prostate cancer.

Bispecific antibodies (BsAbs) are chemically linked or genetically engineered fusion products of either whole or Fab´ fragments of 2 monoclonal antibodies (mAbs). MDX-H210 is constructed from the murine mAb 520C9, which recognizes HER2, and the humanized murine mAb H22, which recognizes the high-affinity type I Fc receptor CD64 (Fc$\gamma$R1), present on monocytes/ macrophages and activated neutrophils. In vitro, MDX-H210 effectively induces antibody-dependent cellular cytotoxicity against HER2 positive targets (Fanger et al, 1992). The binding site for MDX-H210 on the CD64 molecule is distinct from that of IgG and there is no competition for the receptor from endogeneous antibody (Guyre et al, 1989). We chose to combine the antibody with the cytokine GM-CSF as preliminary phase I studies had shown the combination to be well tolerated (Posey et al, 1999). Preclinical studies have shown that GM-CSF can augment monocyte anti-tumour function, enhance in vitro monocyte (Metcalf, 1986) and granulocyte (Kushner and Cheung, 1989) antibody-directed cellular cytotoxicity, and increase expression of CD64 (Connor et al, 1990).

We have previously reported preliminary results using an intermittent schedule of treatment with a 3-week ‘rest period’ after each 3 week of dosing period (James et al, 1998). This trial showed that a number of patients had initial PSA responses but that PSA values rose during the rest period. The treatment was well tolerated. The present study reports results using a continuous dosing schedule in a similar patient group. The primary objective of the study was to assess the activity of the combination of

MDX-H210 and GM-CSF and to evaluate the safety and tolerability of the combination with continuous treatment.

### PATIENTS AND METHODS

### Patients

Patients with hormone refractory adenocarcinoma of the prostate were eligible for inclusion. A maximum of 2 prior regimens of hormonal manipulation (drug therapy or surgery) for treatment of metastatic disease were permitted, with orchidectomy counting as 1 of the 2 endocrine manipulations. All prior radiation therapy and surgery must have been completed at least 6 weeks before protocol therapy and the patient fully recovered from any toxicity. Hormonal treatment was continued (e.g., Lupron) except for flutamide or bicalutamide which were discontinued for at least 6 weeks prior to MDX-H210 therapy. Patients were evaluated for a withdrawal response 6 weeks after cessation of non-steroidal anti-androgen perior to entering the study.

Tumour biopsies had to show overexpression of the HER2 as detailed below. Other eligibility criteria included: adequate haematological, hepatic, and renal function, > 18 years of age, ECOG performance status 0 or 1, life expectancy > 6 months, and serum PSA >50 ng $ml^{-1}$ or <50 ng $ml^{-1}$ and rising on 3 separate occasions over the previous 12 months. All patients gave written informed consent.

Baseline investigations included imaging as indicated by disease state including bone scan on all patients, quality of life assessment and pain scores, 12 lead ECG, full blood count, biochemical profile, coagulation profile, urinalysis, human anti-bispecific antibody (HABA) levels and serum PSA levels.

### Immunohistochemistry

Immunohistological staining for HER2 was done on formalin-fixed, paraffin wax-embedded tumour. These were usually sections of material taken at the time of original diagnosis, often years before treatment. Sections were boiled in 0.01 M citrate buffer pH 6.0 for 30 minutes in a microwave oven. Endogenous peroxidase was blocked with 0.5% hydrogen peroxide in methanol. Rabbit antiserum to HER2 (Dako) was used at 1:25. Biotinylated goat antiserum to rabbit immunoglobulins (Dako Duet) at 1:100 and streptavidin-biotin-peroxidase complex (Dako Duet) at 1:100 were the second and third stages. Peroxidase was detected with 1% tetra-aminobiphenyl hydrochloride (diaminobenzidine) with 0.01% hydrogen peroxide. Positive staining was defined as staining of the cell membrane of most viable cells in the cancer. Sections could be separated into unequivocally positive or unequivocally negative. A control section of carcinoma with positive staining was included in every batch.

### Treatment

Patients were treated with GM-CSF (Monograstim, Schering Plough in the UK or Leukine, Immunex in the USA) 5 µg $kg^{-1}$ $day^{-1}$ by subcutaneous injection for 4 days plus MDX-H210 15 mg $m^{-2}$ by intravenous infusion on day 4, repeated weekly for 6 weeks. Patients were reassessed during the final week of each cycle. Patients with stable disease or better were eligible for further cycles of treatment. A dose modification schedule was included in the protocol but no patient required dose modification during the study period.

### Pain scores

Pain scores were assessed using the Wisconsin Brief Pain Questionnaire which uses a scale from 0 ('no pain at all') to 10 ('pain as bad as you can imagine'). Patients with evaluable pain had to have an average score ≥1 for 1 cycle.

### Response criteria

Response assessment in prostate cancer is problematical as patients rarely have objectively assessable tumour masses. Response to treatment was assessed at the end of each 6 week cycle. A pain response was defined as a mean 2-point decrease or 1-point if baseline score was 1. A PSA response was defined as ≥50% fall from baseline which has been shown to be of prognostic significance (Smith et al, 1998) and is felt by most investigators to be clinically meaningful (Dawson, 1998). Toxicity was assessed using the NCI Clinical Toxicity Grading (CTG) system. In addition, changes in PSA velocity (Vollmer et al, 1998) in the pre study and on study period were compared where adequate pre study PSA data were available.

### Statistical considerations

Patients completing one cycle of treatment (6 weeks) were eligible for the efficacy analysis. All patients receiving treatment were included in the toxicity analysis. Data was censored on 25 November 1998. Response and toxicity data were analysed with simple descriptive statistics. In addition, PSA relative velocity was analysed using the formula of Vollmer et al (Vollmer et al, 1998).

Survival curves were calculated according to the method of Kaplan and Meier (Kaplan and Meier, 1958) and the log-rank test (Peto et al, 1977) used to assess differences between response groups.

### RESULTS

### Patient characteristics

25 patients entered the trial and are included in the safety analysis. Mean age was 65 (range 55–79) years. Prior therapy included palliative chemotherapy (6 patients), radiotherapy (9 patients) and radical prostatectomy (3 patients). Patient characteristics, including prior therapy are shown in Table 1. Overall, 76% of patients screened were HER2 positive. Tumours fell into 2 groups and with either all or nearly all cells positive or none or hardly any cells positive.

### Toxicity

The majority of patients experienced only grade 1–2 toxicity, principally flu-like symptoms such as low-grade fevers, myalgia and sweats. These were generally self-limiting and either required no therapy or simple analgesia such as paracetamol. There were 10 adverse events of grade 3 or 4 rated as definitely, probably or possibly due to the treatment (Table 2). There were no treatment-related deaths. 5 patients completed less than 1 cycle of treatment; 1 for personal reasons, 1 developed heart failure on commencing

© 2001 Cancer Research Campaign

**Table 1** Patient characteristics and prior therapy

| | |
|---|---|
| Age (y) Median (range) | 67.0 (54–78) |
| Race | |
| Caucasian | 22 (88.0%) |
| Black | 2 (8.0%) |
| Hispanic | 1 (4.0%) |
| ECOG performance status | |
| 0 | 17 (68.0%) |
| 1 | 7 (28.0%) |
| 2 | 1 (4.0%) |
| Duration of cancer diagnosis median (range) | 2.70 (0.8–9.4) y |
| Prior systemic cancer therapy: | |
| None | 1 (4.0%) |
| Yes | 24 (96.0%) |
| Chemotherapy | 6 (25.0%) |
| Hormonal | 23 (95.8%) |
| Immunotherapy | 0 |
| Other | 6 (25.0%) |
| Prior radiation: | |
| None | 9 (36.0%) |
| Yes | 16 (64.0%) |
| Prior surgery: | |
| None | 3 (12.0%) |
| Yes | 22 (88.0%) |

GM-CSF and did not receive MDX-H210; 2 patients had severe allergic reactions (anaphylactic and bronchospasm) and 1 left for disease progression after 2 weeks. These patients are not included in the analysis of efficacy.

There was no relationship between HABA titres and response or toxicity. Maximum toxicity with infusion of MDX-H210 generally occurred with the first infusion (with one exception, see Table 1), with little or no toxicity on rechallenge, a phenomenon also observed with other antibody therapies (Cobleigh et al, 1998; McLaughlin et al, 1998; Slamon et al, 1998) and previously reported by us with the intermittent antibody infusion schedule (James et al, 1998).

### Treatment efficacy

All patients included in the efficacy analysis completed at least 1 cycle of treatment. 7 patients of 20 (35%) evaluable patients had a PSA reduction of >50%, ranging from 51% to 99%, with duration of PSA reductions of 50% or greater from entry into the study at the censor date being 71, 83, 89, 122, 128, 160+ and 184+ days. In addition, a further 6 patients experienced minor PSA responses (<50%, >25% reduction in PSA) of 41, 89+, 131, 140, 152 and 165 days duration. Patients with less than 25% reduction in PSA or progression <25% from baseline remained on treatment for 23, 27, 29, 38, 53 and 141+ days. 6 of 12 (50%) patients with evaluable pain have had improvements in pain scores (7–4, 6–0, 4–1, 1–0, 1–0, 1–0). All had reductions in PSA of >25%. Responses lasted for 1 cycle (1 patient), 2 cycles (2 patients), 3 cycles (2 patients), 4 cycles (1 patient) and 12 cycles (1 patient). No patient developed new bone lesions on treatment. Withdrawal was for PSA progression. No patient had complete resolution of bone lesions. Treatment was discontinued in 17 patients for progression, 3 for toxicity, 2 refused further treatment, 2 for other reasons. One patient continued on treatment at the censor date.

The PSA relative velocity was calculated for the period prior to study entry and for the period on study (Vollmer et al, 1998) and is shown in Figure 1. The PSA velocity on therapy decreased in 15/18 (83%) patients with sufficient pre-study PSA data (paired *t*-test for pre-treatment vs. post-treatment PSA relative velocity *P* (two tailed) = 0.0006). This effect was most marked in the responding patients.

### DISCUSSION

Patients with hormone-refractory prostate cancer are among the most challenging to treat in oncology. In this setting, MDX-H210 plus GM-CSF has shown clinical benefit, with a highly acceptable therapeutic index and this study we believe is amongst the first evidence of clinical responses to immunotherapy in prostate cancer. We had in addition seen PSA responses >50% in 5/18 patients treated with an intermittent schedule of the 2 drugs (3 weeks on, 3 weeks off). In that study, a proportion of patients experienced a rise in PSA in the rest period, but little toxicity on treatment. Treatment-related toxicity in the first study was predominantly grade 0–2 with patients able to tolerate prolonged dosing as in the present study (James et al, 1998). Accordingly, it was decided to proceed with the continuous therapy regimen described in this paper. Similar results have been reported using a slightly different GM-CSF schedule (Small et al, 1999) but only 1 patient experienced a PSA fall >50% sustained for >6 weeks. In contrast, an immunotherapy study by Slovin et al of an $^{131}$I-labelled anti-TAG72 plus interferon-γ failed to show any activity (Slovin et al, 1998).

The results clearly demonstrate a high frequency of clinically significant reductions in PSA that were durable in nature. 35% of the patients treated had greater than 50% reductions in PSA levels in response to treatment. 63% of patients continued to be treated for 77 days or more without disease progression or with continued reductions in PSA levels. No patient developed clinical features of disease progression whilst the PSA level had fallen below baseline. In addition, as can be seen from Figure 1, the average PSA velocity during the period on therapy decreased in all except for 3 patients. All patients with pain score improvements had falls in PSA of at least 25%. Patients remained on treatment until either disease progression or toxicity resulted in withdrawal.

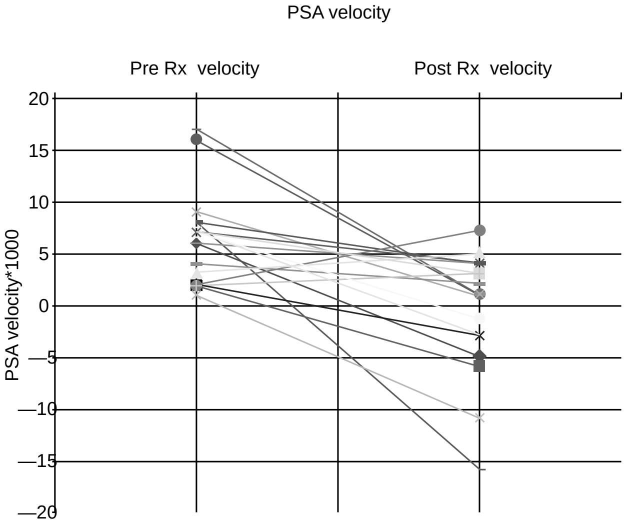

**Figure 1** PSA velocity calculated for the period prior to commencing treatment and compared to the period on treatment with GM-CSF and MDX-H210.

 *© 2001 Cancer Research Campaign*

**Table 2** Severe adverse events considered possibly, probably, or definitely related to treatment

| Symptom | Patient | Cycle | Onset day | Res. day | Grade | Action | Outcome |
|---|---|---|---|---|---|---|---|
| Allergic reaction | A | 1 | 18 | 18 | 3 | DC’d | Rec |
| Asthma | B | 1 | 4 | 5 | 3 | None | Rec |
| Chills | C | 1 | 4 | 4 | 3 | Inter | Rec |
| | D | 3 | 117 | 117 | 3 | Inter | Rec |
| | E | 1 | 4 | 4 | 3 | Inter | Rec |
| Heart failure | F | 1 | 1 | 8 | 4 | DC’d | Rec |
| Dyspnoea | G | 1 | 4 | 4 | 3 | Inter | Rec |
| | H1 | 4 | 4 | 4 | DC’d | Rec | |
| Headache | G | 2 | 48 | 48 | 3 | None | Rec |
| | G1 | 1 | 1 | 3 | None | Rec | |
| Hypertension | I | 1 | 4 | 4 | 3 | None | Rec |
| Pain | J | 2 | 57 | 57 | 3 | None | Rec |

Onset day = onset day relative to first day of GM-CSF; Res. day = resolution day relative to first day of GM-CSF; Grade = toxicity grade according to NCI toxicity criteria; Action: Inter = regimen interrupted; DC’d = drug discontinued; Outcome: Rec = recovered.

The quality of life evaluation revealed that two-thirds of those treated either had significant improvement in their pain, had no new pain if there was no pain at baseline, or had no worsening of their pain. The treatment regimen was well tolerated, with only 2 patients withdrawing from the study because of adverse events, one due to GM-CSF and one due to MDX-210 but associated with underlying disease progression.

The combination regimen evaluated was initially designed because of evidence of an in vitro interaction between the bispecific antibody and GM-CSF (Fanger et al, 1992), so the combination with the antibody seemed logical to maximize the possibility of clinical response. Since the trial commenced, studies have reported evidence of direct clinical activity for GM-CSF in prostate cancer (Simons et al, 1998; Small et al, 1999). The results of the combination are thus in part due to GM-CSF and we are currently carrying out a phase II study to assess the activity of the antibody alone.

In conclusion, evidence is presented of clinical responses in patients with hormone refractory prostate carcinoma. Toxicity was generally mild to moderate and mostly manageable on an outpatient basis. Further studies of regimens containing MDX-H210 and GM-CSF are indicated in prostate cancer.

### REFERENCES

Cobleigh MA, Vogel CL, Tripathy D, Robert NJ, Scholl S, Fehrenbacher L, Paton V, Shak S, Lieberman G and Slamon D (1998) Efficacy and safety of Herceptin (humanized anti-her2 antibody) as a single agent in 222 women with her2 overexpression who relapsed following chemotherapy for metastatic breast cancer. *Proc of ASCO* **17**: 97a

Connor RI, Shen L and Fanger MW (1990) Evaluation of the antibody-dependent cytotoxicity capabilities of individual human monocytes – role of Fc gamma R1 and Fc gamma R2 and the effects of cytokines at the single cell level. *J Immunol* **145**: 1483–1489

Craft N, Shostak Y, Carey M and Sawyers CL (1999) A mechanism for hormone-independent prostate cancer through modulation of androgen receptor signaling by the HER-2/neu tyrosine kinase [see comments]. *Nature Medicine* **5**: 280–285

Dawson NA (1998) Apples and oranges: building a consensus for standardized eligibility criteria and end points in prostate cancer clinical trials [see comments]. *J Clin Oncol* **16**: 3398–3405

Fanger MW, Morganelli PM and Guyre PM (1992) Bispecific antibodies. *Critical Rev Immun* **12**: 101–121

Fox SB, Persad RA, Coleman N, Day CA, Silcocks PB and Collins CC (1994) Prognostic value of c-erbB-2 and epidermal growth factor receptor in stag A1 (T1) prostatic adenocarcinoma. *British Journal of Urology* **74**: 214–220

Guyre PM, Graziano RF, Vance BA, Morganelli PM and Fanger MW (1989) Monoclonal antibodies that bind to distinct epitopes on Fc gamma RI are able to trigger receptor function. *J Immun* **143**: 1650–1655

James N, Atherton P, Koletsky A, Tchekmedyian N and Curnow R (1998a) Phase II trial of the bispecific antibody MDX-H210 (anti-HER2/neu x anti-CD64) combined with GM-CSF in patients with advanced prostate and renal cell carcinomas that express HER2/neu. *B J C* **78**: 56

James N, Atherton P, Koletsky A, Tchekmedyian N and Curnow R (1998b) Phase II antibody trial of the bispecific antibody MDX-H210 (anti-HER2/neu x anti-CD64) combined with GM-CSF in patients with advanced prostate and renal cell carcinomas that express HER2/neu. *ProcASCO* **17**:

Kallakury, BVS, Sheehan C, Ambros RA, Fisher HA, Kaufman JrRP, Muraca PJ and Ross JS (1998) Correlation of p34cdc2 Cyclin-Dependent Kinase Overexpression, CD44s Downregulation, and HER-2/neu in Prostatic Adenocarcinomas. J Clin Oncol **16**: 1302–1309

Kaplan EL and Meier P (1958) Non-parametric estimation from incomplete observations. *J Amer Stat Assoc* **53**: 457(Abstract)

Kushner BH and Cheung NK (1989) GM-CSF enhances 3F8 monoclonal antibody-dependent cellular cytotoxicity against melanoma and neuroblastoma. *Blood* **73**: 1936–1941

Lyne JC, Melhem MF, Finley MDGG, Wen D, Liu PhDN, Deng BSDH and Salup R (1997) Tissue Expression of Neu Differentiation Factor/Heregulin and Its Receptor Complex in Prostate Cancer and Its Biologic Effects on Prostate Cancer Cells in Vitro. *Cancer J Scientific Amer* **3**: 21–30

McLaughlin P, Grillo-Lopez AJ, Link BK, Levy R, Czuczman MS, Williams ME, Heyman MR, Bence-Bruckler I, White CA, Cabanillas F, Jain V, Ho AD, Lister J, Wey K, Shen D and Dallaire BK (1998) Rituximab chimeric anti-CD20 monoclonal antibody therapy for relapsed indolent lymphoma: half of patients respond to a four-dose treatment program. *J Clin Oncol* **16**: 2825–2833

Metcalf D (1986) The biology and function of the granulocyte macrophage colony stimulating factors. *Blood* **67**: 257–267

Morote J, de T I, Caceres C, Vallejo C, Schwartz SJ and Reventos J (1999) Prognostic value of immunohistochemical expression of the c-erbB-2 oncoprotein in metastasic prostate cancer. *Inter J Cancer* **84**: 421–425

Peto R, Pike MC, Armitage P, Breslow NE, Cox DR, Howard SV, Mantel N, McPherson K, Peto J and Smith PG (1977) Design and analysis of randomized clinical trials requiring prolonged observation of each patient. II. analysis and examples. *B J C* **35**: 1–39

Posey JA, Raspet R, Verma U, Deo YM, Keller T, Marshall JL, Hodgson J, Mazumder A and Hawkins MJ (1999) A pilot trial of GM-CSF and MDX-H210 in patients with erbB-2-positive advanced malignancies. *J Immuno* **22**: 371–379

Ross JS, Nazeer T, Church K, Amato C, Figge H, Rifkin MD and Fisher HAG (1993) Contribution of HER-2/neu oncogene expression to tumor grade and DNA content analysis in the prediction of prostatic carcinoma metastasis. *Cancer* **72**: 3020–3028

Ross JS, Sheehan BC, Hayner-Buchan MAM, Ambros MRA, Kallakury MBVS, Kaufman MR, Fisher MHAG, Rifkin MMD and Muraca BPJ (1997a) Prognostic significance of HER-2/neu gene amplification status by fluorescence in situ hybridization of prostate carcinoma. *Ameri Cancer Soc* 2162–2170

*© 2001 Cancer Research Campaign* 

Ross JS, Sheehan C, Hayner-Buchan AM, Ambros RA, Kallakury BVS, Kaufman R, Fisher HAG and Muraca PJ (1997b) HER-2/neu Gene Application status in prostate cancer by fluorescence in situ hybridization. *Human Pathology* **28**: 827–833

Sadasivan R, Morgan R, Jennings S, Austenfeld M, Van Veldhuizen PV, Stephens R and Noble M (1993) Overexpression of HER-2/neu may be an indicator of poor Prognosis in prostate cancer. *J Urol* **150**: 126–131

Simons JW, Carducci MA, Weber CE, De Marzo A, Baccala A, Cohen L, Clift SM, Mikhak B, Piantadosi S, Partin AW, Carter HB and Levitsky HI (1998) Bioactivity of autologous irradiated prostate cancer vaccines generated by *ex vivo* gm-csf gene transfer. *Proc ASCO* **17**: Abstract 1205

Slamon D, Leyland-Jones B, Shak S, Paton V, Bajamonde A, Fleming T, Eiermann W, Wolter J, Baselga J and Norton L (1998) Addition of HerceptinÖ (humanized anti-her2 antibody) to first line chemotherapy for her2 overexpressing metastatic breast cancer (her2+/mbc) markedly increases anticancer activity: a randomized, multinational controlled phase III trial. *Proc ASCO* **17**: 98a

Slovin SF, Scher HI, Divgi CR, Reuter V, Sgouros G, Moore M, Weingard, Pettengall R, Imbriaco M, El-Shirbiny A, Finn R, Bronstein J, Brett, Milenic D, Dnistrian A, Shapiro L, Schlom J and Larson SM (1998) Interferon-gamma and monoclonal antibody 1311-labeled CC49: Outcomes in patients with androgen-independent prostate cancer. *Clin Cancer Res* **4**: 643–651

Small EJ, Reese DM, Um B, Whisenant S, Dixon SC, Figg and WD (1999) Therapy of advanced prostate cancer with granulocyte macrophage colony-stimulating factor. *Clin Cancer Res* **5**: 1738–1744

Smith DC, Dunn RL, Strawderman MS and Pienta KJ (1998) Change in serum prostate-specific antigen as a marker of response to cytotoxic therapy for hormone-refractory prostate cancer. *J Clin Oncol* **16**: 1835–1843

Vollmer RT, Dawson NA and Vogelzang NJ (1998) The dynamics of prostate specific antigen in hormone refractory prostate carcinoma. *Cancer* **83**: 1989–1994

Yeh S, Lin HK, Kang HY, Thin TH, Lin MF and Chang C (1999) From HER2/Neu signal cascade to androgen receptor and its coactivators: a novel pathway by induction of androgen target genes through MAP kinase in prostate cancer cells. *Proc Nat Acad Sci USA* **96**: 5458–5463

 *© 2001 Cancer Research Campaign*
